# Adverse events following immunization reporting and impact on immunization services in informal settlements in Nairobi, Kenya: a prospective mixed-methods study

**DOI:** 10.11604/pamj.2021.40.81.25910

**Published:** 2021-10-07

**Authors:** Oliver Ombeva Malande, Deogratias Munube, Rachel Nakatugga Afaayo, Carolyne Chemweno, Mutunga Nzoka, James Kipsang, Andrew Munyalo Musyoki, Johanna Catharina Meyer, Leonidah Nyamusi Omayo, Lawrence Owino-Okongo

**Affiliations:** 1 East Africa Centre for Vaccines and Immunization, Administration Department Kampala, Kampala, Uganda,; 2 Department of Paediatrics and Child Health, Egerton University, Nakuru, Kenya,; 3 Department of Paediatrics and Child Health, Makerere University, Kampala, Uganda,; 4 Division of Public Health Pharmacy, Sefako Makgatho Health Sciences University, Pretoria, South Africa,; 5 Department of Microbiological Pathology, Sefako Makgatho Health Sciences University, Pretoria, South Africa,; 6 Department of Paediatrics and Child Health, University of Nairobi, Nairobi, Kenya

**Keywords:** Vaccination, immunization programs, focus groups, child, informal settlements, Kenya

## Abstract

**Introduction:**

adverse events following immunization (AEFIs) are thought to contribute to cases of vaccine hesitancy, yet little data exists describing the state of reporting and management of AEFIs. This study investigated the occurrence and influence of AEFIs on vaccine hesitancy in an informal settlement of Nairobi.

**Methods:**

this was a prospective mixed-methods study involving 7 focus group discussions, 8 key informant interviews and 457 face-to-face interviews with caregivers. Caregivers were recruited at/or before the 6 week clinic visit and assessed for occurrence of AEFIs in their children at the subsequent 10- and 14-week visits and a follow-up two weeks following the 14 weeks visit via phone calls.

**Results:**

in this study, 12.3% (56/457) of the infants experienced an AEFI. Of these, 19 did not report for the next scheduled vaccine. Fever was the most common AEFI, for which most caregivers (66.7%) used Paracetamol as antipyretic, while 20.8% sought help from a nearby health facility. Three of the 56 AEFIs (convulsions) that occurred in study participants could be classified as severe reactions. Diphtheria, pertussis and tetanus (DPT 3) completion rate was 75.3%. Most (96.4%) caregivers considered immunization an important strategy for child survival. Vaccine hesitancy occurred among 3.6% of participants, 30% of whom attributed their hesitancy to occurrence of AEFIs. The review of health records revealed that no AEFI had been reported from any of the study facilities.

**Conclusion:**

cases of adverse events following immunization are not reported in Mathare Valley and they do have implications for vaccine hesitancy by some caregivers.

## Introduction

The significant contribution of vaccine preventable diseases (VPDs) to morbidity and mortality in developing countries means that immunization remains a reliable child survival strategy. Childhood immunization is estimated to prevent over 2.5 million child deaths each year [1-4]. While there is appreciable global progress to ensure provision of childhood vaccinations, difficulties exist particularly regarding best approaches to reach the more vulnerable, remote, poorest and disadvantaged child populations in sub-Saharan Africa [5-7]. These populations are affected by several factors that reduce access to and uptake of vaccines including teenage or advanced age of caregivers who may not effectively adhere to the immunization schedule, difficult transport and geographical terrain that affects accessibility to immunization centres, large refugee and immigrant populations that tend to be highly mobile, increasing cases of negative messages and anti-vaccine sentiments, poor socioeconomic status, and fear of possible adverse reactions after vaccination [3, 5-8].

While all the currently recommended vaccines are considered safe and effective, these vaccines may occasionally lead to undesirable effects referred to as Adverse Events Following Immunization (AEFIs). AEFIs may reduce public confidence in immunization, leading to reduced uptake of childhood immunizations and increased dropout rates, thus slowing down the gains made in ensuring universal access to all vaccines by all vulnerable populations [8-10]. Cognizant of vaccine safety concerns, the World Health Organization (WHO) and a group of other partners launched the Global Vaccine Safety Initiative (GVSI) to provide a framework for enhancing vaccine safety and pharmacovigilance in all countries [11]. AEFIs may be classified according to seriousness and severity. An AEFI is classified as serious if it results in death, is life-threatening, requires in-patient hospitalization, results in significant incapacity, or results in a congenital anomaly. The severity of AEFIs refers to the intensity of the reaction and can be classified as minor or severe events. Severe vaccine reactions include seizures, prolonged crying, disseminated *Bacillus Calmette–Guérin* (BCG) disease, anaphylaxis and vaccine associated paralytic polio. Minor vaccine reactions include fever, pain, redness at the injection site and crying and are not obligatory reportable events in most countries including Kenya. Both the serious and severe AEFIs warrant reporting to the authorities.

The Kenya Demographic Health Survey (KDHS) 2014 found that the fully immunized under 1-year-old coverage (FIC) rate was 71%; implying that only 71% of the Kenyan children received all the basic immunizations they were eligible for by the age of one year [12]. However, there is great disparity in coverage by regions and even within regions, with informal settlements being identified as some of the areas with the lowest vaccination coverage [13, 14]. Rumours about, and fears of AEFIs, have been identified as some of the factors that contribute to failure to take children for immunization and for immunization dropout [10, 11]. Lack of information provided to caregivers and lack of avenues to report AEFIs when they occur, may contribute to the misinformation about vaccine safety [10, 11, 13]. Even though the Expanded Program on Immunization (EPI) in Kenya was started in 1980, there are only a few cases of AEFIs reported and documented at national level to date [15]. To the best of our knowledge, it is not clear whether the problem is reporting itself, or documentation of AEFIs by either the caregivers or healthcare workers (HCWs). Furthermore, there is limited information about the contribution of AEFIs towards failure to complete the immunization schedule in Kenya.

This study sought to determine the occurrence and influence of AEFIs towards failure to complete the immunization schedule by employing the use of mobile phones in the follow-up of caregivers who missed their scheduled visits in an informal settlement in Nairobi. The study also sought to determine the knowledge, experience, perceptions, and practices regarding AEFI reporting and surveillance among health workers serving an informal settlement in Nairobi.

## Methods

**Study design and setting:** this was a prospective mixed-methods study conducted at 10 immunizing health facilities in Mathare Valley, utilizing both qualitative and quantitative research approaches. Mathare Valley is an informal settlement, which straddles Starehe and Ruaraka sub-counties of Nairobi, with an estimated population of over 200,000 people living in an area of 0.8 square kilometres [12]. It has poor supply of electricity, running water and sanitation facilities. Most of the inhabitants live on an income of less than a dollar per day. Immunization services are provided by facilities in the area and in the periphery [12]. The entire data collection, facility visits, interviews and focus group discussions were supervised by the principal investigators and co-authors, who were the study collaborators, in strict adherence to study protocol, following set standards and conditions. Sample size was estimated by use of the World Health Organization´s (WHO) 30 cluster sampling technique for cluster survey design. A multi-stage cluster sampling method was used that involved all the locations within the two main sub-counties. A sub location constituted a cluster to ensure a wide distribution across the two sub-counties.

Qualitative data were collected through Focus Group Discussions (FGDs) conducted with child caregivers and Key Informant Interviews (KIIs) with immunization program managers, HCWs and other key stakeholders. Quantitative data were collected through face-to-face interviews with caregivers presenting children for their 6-week vaccination, with prospective follow-up at the subsequent 10 and 14 weeks vaccination visits. At the time of the study, the immunization schedule in Kenya included administration of oral poliovirus vaccines (OPV), DPT-Hib-HepB and pneumococcal vaccines at 6, 10 and 14 weeks; as well as oral Rota virus vaccine at 6 and 10 weeks. Mobile phones were used to follow-up on all caregivers 1-2 weeks after the 14 weeks vaccination to determine occurrence of any AEFIs and to trace defaulter caregivers who missed their children´s scheduled visit. Secondary data on AEFIs reported over the previous year were collected from the study facilities, sub-county managers and from the EPI national office.

**Data collection and sample population:** all quantitative data were collected by a team of well-trained research assistants between August and November 2017. Each data collection team consisted of 4 trained research assistants. Face-to-face interviews were conducted with caregivers, being the first and last caregiver receiving the immunization services, and selected purposively.

**Follow-up component of the study:** for the prospective component of the study, consecutive sampling was used to recruit consenting caregiver-child pairs, (i.e. a caregiver with a child who has come for the 6-week immunization visit) until the desired sample size of 457 caregiver-child pairs was attained. Caregivers who failed to provide informed consent and those who had been residents in the Mathare Valley for less than three months were excluded. Caregiver-child pairs were followed-up at the 10-week and 14-week immunization visits, as well as a phone interview 1-2 weeks after the 14-week visit.

Demographic details of the child and caregiver, including age, sex, educational level of caregiver, area of residence, maternal occupation and vaccine administered were collected using a pretested interview administered questionnaire. During follow-up visits, research assistants asked whether the child had experienced any undesired effect following the immunization; and if any, what these undesired effect(s) were and what action was taken by the caregiver. Research assistants also asked for reasons of defaulting on the immunization schedule. A follow-up call was made after one week of missing the appointment to any caregiver whose child failed to attend a scheduled immunization visit at 10 and 14 weeks in order to establish reasons for defaulting, to determine if there were any AEFIs associated with the 6, 10 and 14-week vaccination and to encourage the caregiver to take the child for the scheduled vaccination. Children were followed-up till they completed the 14-week vaccination visit, with a final telephone call made to all caregivers, 1-2 weeks after the 14-week vaccination, to determine if there were any AEFIs associated with the vaccines administered. Caregivers were provided with a toll-free mobile phone number to contact the study personnel to report any untoward events following vaccination. When such a report was received, caregivers were advised on measures to take to manage the AEFIs, and to report to the facility for evaluation if indicated.

**Focus groups discussions and key informant interviews:** all FGDs and KIIs were conducted at community halls, churches and schools; not in the health facilities. The FGDs and KIIs were audio-recorded and conducted in Kiswahili by a well-trained moderator, with a research assistant taking detailed notes. Participants for FGDs with caregivers were recruited through purposive sampling in order to achieve maximum variation in the sample (Table 1). Seven FGDs (one FGD per health facility) were conducted, each group consisting of between 8-12 participants, with a total number of 71 participants from the 7 groups. The duration of FGDs were between 60-80 minutes, with saturation of data achieved for all themes identified. Participants for KIIs and face-to-face interviews were selected purposively. KIIs included nurses who had been working for at least 3 months in health facilities that offer immunization services to children residing in Mathare Valley, and other immunization program managers.

**Table 1:** focus group discussion guide

Why do you think children are given vaccines/immunization? What kind of diseases can vaccines prevent?
**Were there situations when you failed to bring your child for immunization?**
Probe: what were the reasons?
Probe: how are women who take their children for vaccination in health facilities treated by the health workers?
**Do you think that it is difficult for some ethnic or religious groups in your community/region to get vaccinations for their children?**
Probe: if yes, what do you think are the reason(s)?
**Do you think that most parents from your area accept taking their children for immunization?**
Probe: are there those who do not?
Probe: what are some of the reasons why they opt not to take the children for immunization?
**Are there any reasons you can think of why children should not be vaccinated?**
Probe: if yes, please specify
**Are there days you went to a health facility and found when there were no vaccines?**
Probe: which vaccine was it?
Probe: and what did you do to get your child vaccinated?
**Have you ever received or heard negative information about vaccinations?**
Probe: if yes, please give an example
**Did you still take your child to get vaccinated after you heard the negative information?**
Probe: are there any side effects to vaccines?
Probe: has your child or a child you know of ever got those side effects?
Probe: what did the parent or caretaker do to help the affected child?
**Do leaders (religious or political leaders, teachers, health care workers) in your community support vaccines for infants and children?**
**Are there any religious groups or cultural groups you know of, (maybe not from your area) that do not encourage or promote immunization for children?**
Probe: if yes, what are their reasons for being against vaccines/immunization?
**What is the first thing the government does when they want to introduce a new vaccine to your area?**
Probe: do they educate the community enough?
Probe: do they usually get feedback from the community?
**Are there any reactions or problems or adverse reactions that occur following vaccinations, whether in your child or in another child you know of?**
Probe: how do you or did the affected caregiver handle this problem?
Probe: what in your view is the best and correct way to handle such a problem?

**Ethical considerations:** ethical approval was obtained from the Kenyatta National Hospital/University of Nairobi Ethics and Research committee (KNH-UON ERC Ref. KNH-ERC/A/158]. Separate individual written informed consent was obtained from each participant interviewed, namely FGD participants, key informants and parents/caregivers of all minors. Where additional information was sought or required that was not covered by any of these levels of consent, especially for secondary data, the need for such consent was waived/granted by the Kenyatta National Hospital/University of Nairobi Ethics and Research committee [KNH-UON ERC Ref. KNH-ERC/A/158]. Personal identifiers were delinked from all participant responses. All data and the information collected in this study are safely stored.

**Data management:** audio-recorded FGDs and KIIs were transcribed verbatim by the research assistants who were fluent in both Kiswahili and English using Microsoft Word®. Each audio recording was compared to its respective transcript and notes taken for accuracy. Transcripts were subsequently imported into NVivo®12 software for analysis. The focus group moderator and an assigned member of the research team used open coding to code the transcripts. Continuous discussions between them facilitated the development of the coding frame and transition from open codes to themes and sub-themes. Both anticipated and emergent themes were explored. All collected quantitative data were entered without recording any personal identifiers in a Microsoft Excel® 2016 spreadsheet and then exported to Stata® version 13 for analysis. Descriptive data are expressed as means ± standard deviation, frequencies and percentages.

## Results

### Face-to-face interviews with caregiver/child pairs

**Socio-demographic characteristics:** this study successfully enrolled and conducted face-to-face interviews with 457 caregiver/child pairs. From the 457 caregiver/child pairs enrolled, the majority (92%; 419/457) were female, predominantly Protestants 66% (x/457) and 82% (374/457) were married. Other caregiver characteristics are shown in Table 2.

**Table 2:** characteristics of caregivers

Variable	Frequency (N=457)	Percentage (%)
**Relationship to the participant child**		
Parent	419	91.6%
Others	38	8.3%
**Age**		
≤25	301	65.9%
26-30	114	25.5%
31-35	27	6.2%
36-40	13	3.0%
>40	2	0.4%
**Sex**		
Male	38	8.0%
Female	419	92.0%
**Religion**		
Muslim	30	6.0%
Catholic	79	18.1%
Protestant	298	66.3%
Seventh Day Adventist	50	10.0%
**Marital status**		
Married	374	81.8%
Single	51	11.1%
Widowed	25	5.6%
Divorced	7	1.5%
**Occupation**		
Unemployed	83	18.2%
Unskilled labor	296	64.8%
Skilled labor	78	17.0%
**Education level**		
None	4	1.0%
Primary	297	65.0%
Secondary	123	27.7%
Tertiary	3	6.3%
**Caregiver being the household head**		
No	338	74%
Yes	119	26%
**Sex of the household head**		
Male	360	78.8
Female	97	21.2

**Figure 1:**
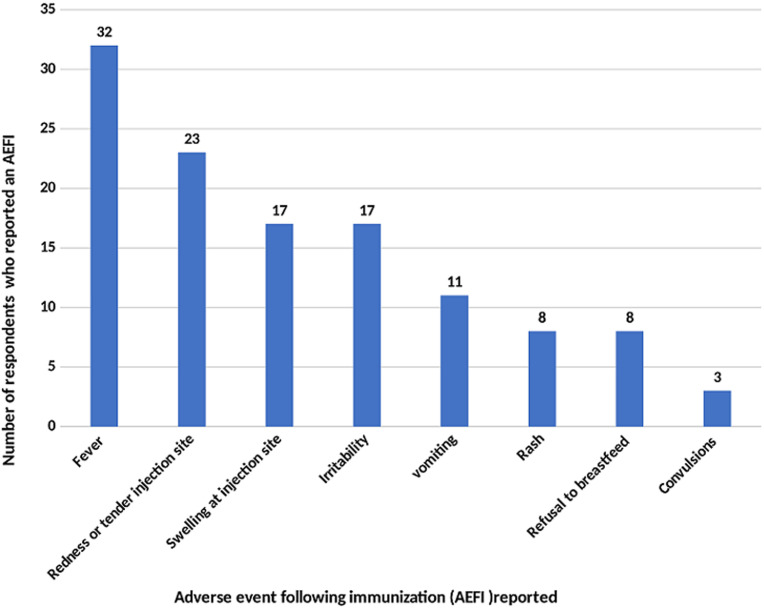
adverse events following immunization reported by respondents (n=56)

**Adverse events following immunization:** all 457 infants were enrolled at the 6-week immunization visit, and followed-up at the 10- and 14-weeks immunizations and again 1-2 weeks after the 14-week immunization visit. The Pentavalent 3 (14-week) immunization completion rate was 75.3%. Only 12.3% (56/457) experienced an AEFI, the majority (57.1%; 32/56) of whom experienced fever post vaccination and 33.3% (19/56) not reporting for the next scheduled immunization visit. The other reported AEFIs as shown in Figure 1 below were redness or tenderness at the injection site (23/56), swelling at the injection site (17/56), irritability/crying a lot (17/56), vomiting (11/56), rash (8/56), refusal to breastfeed (8/56) and convulsions (3/56).

Regarding caregiver responses to occurrence of an AEFI (n=56), two thirds 66.7% (x/56) resorted to the use of Paracetamol for fever control, while 20.8% (x/56) sought help from a nearby health facility. The majority (68.5% of the 457) of caregivers indicated that they had received information regarding vaccines in general, of whom 64.3% were educated by health care professionals and 2% were educated by family members. While 28.0% (of the 457) of caregivers had received information regarding AEFIs previously, only 2.1% made decisions regarding immunization as influenced by this previous information or advice.

On satisfaction with immunization health services, 98.8% of the 457 caregivers were satisfied. Immunization as a strategy for under 5 child survival received a 96.4% acceptance rate. Vaccine hesitancy occurred among 3.8% (18/457) of participants, 30% of whom attributed their resistance to occurrence of AEFIs. For the caregivers who were hesitant to vaccinate their children, the reasons they gave included the following: fear of paralysis of the child; fear of side effects; the child being sick at the time of the scheduled immunization; lack of information on immunization; fear that the vaccine can affect the child´s growth and development; and fear of death as a result of immunization.

A review of records at the health facilities within Mathare Valley revealed that no AEFI had been reported from any of these facilities in the preceding 12 months. There was little knowledge among HCWs in the study area of the AEFIs to report, where to report them, to whom to report and how (which tool and process) to report them. The system in place for identification and reporting of AEFIs recommended by the Ministry of Health had not been implemented or fully integrated into the health care system.

The use of mobile telephone for soliciting or reporting AEFIs by caregivers was well received by caregivers who were reached by phone. However, only two calls were received, both from caregivers not enrolled in the study but had heard about the project. This was probably because there were no serious adverse events and because the caregivers were being called after 1-2 weeks following immunization to solicit for any AEFI.

#### Focus group discussions and key informant interviews

**Socio-demographic characteristics of participants:** seven FGDs with a total of 71 caregivers were conducted in the Mathare Valley informal settlement. The majority of the participants in the FGDs were married (82%; 58/71), 17% (12/71) single and one was divorced. Regarding the age of the caregivers, the majority (91%; 65/71) were of age ≤30 years, with only 8.5% (6/71) older than 30 years. The majority of respondents were from the Independent African Churches (59%; 42/71), followed by catholic (18%; 13/71), Seventh Day Adventist (10%; 7/71), Anglican Church of Kenya (7%; 5/71) and Muslim (6%; 4/71). Most participants were unskilled labourers (80%; 57/71) and the rest were small business owners (20%; 14/71). In terms of education, the majority of respondents had attained primary education (65%; 46/71), 30% (21/71) had secondary education, while 4% (3/71) and 1% (1/71) had neither post-secondary education nor any education respectively. Eight interviews were conducted with key informants (KIs) who were nurses heading immunization centres in the Mathare Valley.

**Themes on adverse events following immunization:** the findings from the FGDs and the KIIs are presented according to the two main themes that emerged from the data, each underpinned by two sub-themes. Verbatim quotations from participants, presented in italics and enclosed in quotation marks, are used to illustrate the findings. Each quotation is followed by a notation (FGD-P or KI-P) to identify the source of data, the participant and his/her socio-demographic characteristics.

### Theme I: caregivers´ knowledge, attitudes and perceptions towards child immunization

**a) General knowledge of immunization:** from the FGDs it was evident that most of the caregivers were informed about immunization and its importance for children.

***Protection from diseases and disabilities:*** the majority of caregivers had a good understanding that vaccines protect children from diseases and ensured that if the child happens to get sick after vaccination, he/she will develop only a mild form of the disease. Some caregivers indicated that immunization protects against disabilities and to some extent child death.

*“… Vaccination helps in prevention of diseases like being crippled.”* (FGD7-P4; Married, with no education).

On the other hand, there was no consensus on how much protection immunization offers. While some caregivers believed that immunization is a 100% effective, others were of the opinion that immunization does not provide 100% protection for children, thus does not guarantee protection from illness.

***Boost immune system, grow well and live a healthy life:*** some caregivers specified the benefits of immunization in boosting a child´s immune system. A common view of caregivers was that immunization is important for the child´s healthy growth and being able to life a healthy life, as explained by one of the mothers:

*“……. A child who has been immunized has a lot of energy compared to those who have not.”* (FGD4-P10; Married mother with primary education).

***Reduce the impact of disease:*** notably, participants raised concerns with the recent introduction of the measles vaccine at 18 months, which they believed was not important. However, they strongly advocated for creation of awareness amongst parents regarding the benefits of vaccines, especially in terms of severity, should the child get the disease.

*“… Immunization is very important. If children get vaccinated, for example, one for measles, even if the disease comes, it will be mild with no big effect.”* (FGD1-P11; married mother with secondary education).

**b) Barriers to immunization:** participants in the various FGDs identified several reasons why parents refuse to have their children vaccinated, of which many were related to vaccine safety.

***Negative experiences with AEFIs:*** negative experiences with immunization ranged from what caregivers described as their own experiences, which included symptoms such as pain, fever and discomfort, to what they heard from others. Two of the mothers described their experience as follows:

*“… Some vaccinations have a lot of power making the children not to sleep because of pain. It causes a lot of body heat and the injected leg cannot move.”* (FGD 7-P7; married mother with secondary education).

*“… I have not heard from anybody, but I heard it at the hospital. The doctor told me that if the child is given ROTA vaccine and breastfeeds, the tongue can burst because the child vomits so much”*. (FGD1-P11; married mother with secondary education).

**Influence of family beliefs and behaviour:** the influence of family beliefs and behaviour around vaccination, on caregiver´s decision-making to immunize their children, was mentioned in the various discussions. Two mothers described it as follows:

*“… I have a friend who never took his child to vaccination because her mother never believed in vaccination.”* (FGD6-P1; married with post-secondary education).

*“… Some people say that if our parents were never vaccinated and they are alive so even if I don´t take my child for vaccination, nothing will happen.”* (FGD7-P4; Married, with no education).

***Functionality of the immunization program and commitment of caregivers:*** participants also mentioned concerns related to the availability of vaccines and access to the immunization program, and commitment required of caregivers in response to this.

“… When there is strike, one may be forced … to miss vaccination schedule … due to fear of going to private hospitals which they feel are expensive.” (FGD6-P11; married mother with secondary education).

*“… For example, if you go to the hospital, there are long queues, so somebody just decides that she is not going.”*(FGD7-P6; Married mother with primary education).

***Conspiracy theories and distrust:*** conspiracy theories around vaccines being used to prevent pregnancy, children being used for “trials”, at the same time increasing the prevalence of disease, are some examples raised by caregivers, clearly having a negative impact on the decision to vaccinate.

*“… If the child is immunized, she fails to bear children, so this makes them fear … these vaccinations are just brought by the whites and therefore they are using us. The more they bring them, the more many diseases arise … it does not prevent me since even the other day; … I took my child for vaccination and she felt very sick.”* (FGD2-P3; married mother with no formal education).

***Fear of being reprimanded by HCWs:*** there were a number of scenarios where parents indicated that they fear being severely reprimanded by HCWs, and therefore avoided taking their children for immunization. Examples of such cases included a mother having a home delivery, school girls with early pregnancies, missing any scheduled immunization visits and when the mother loses her child´s clinic card, as explained below.

*“… There are reasons like when I take my child for vaccination at the clinic, you are required to have a clinic card and even if you explain the loss of the card maybe through flooding or got burnt, they just insist that you are lazy.”*(FGD7-P8; Single mom with primary education).

**Myths/misconceptions regarding the immunization program:** certain concerns about vaccines and adverse events following immunization, emanating from myths or misconceptions around the safety of immunization, were raised by caregivers, as illustrated below.

*“… Some say that the type of Polio vaccine which has been brought recently is not good and it does more harm…sometimes people fear because some vaccines maybe expired when they are given and the nurse doesn´t tell you, and then the child dies immediately you reach home.”* (FGD2-P5; single with primary education).

***Religious influences:*** religion emerged as influence on parents´ stance towards immunization and a barrier to uptake of vaccination. A mother described these religious influences as follows:

*“… We once heard the Catholic Church say that the immunization (the one which was conducted on ladies) is bad that it will prevent them from giving birth”* (FGD5-P9; Divorcee mother with primary education).

### Theme II: knowledge of AEFIs and the reporting thereof

**a) Previous experience with AEFI by caregivers:** caregivers were aware of the AEFIs, however, some of their thoughts on AEFIs could be classified as misconceptions. Caregivers were able to recall events that happened to their children or reported to them by others. These AEFIs reported ranged from mild effects like fever and discomfort to those requiring amputation, disability and death of a child.

***Dangers of immunization:*** immunization was perceived as dangerous by certain participants, who alluded to these dangers based on experiences within their families or hearsay in the community. Two mothers who participated in two separate FGDs described these serious AEFIs as follows:

*“My brother´s child was immunized and died the following day.”* (FDG4-P11; Married mother with secondary education).

*“… There was once an injection which was done in Kisumu in the children´s arm and…… (speaking slowly) when they woke up the following day, all of them were crippled and the next step was to amputate the affected arms*. (FDG1-P6; Single mother working in a saloon with basic education).

***Fear of possible AEFI:*** experiences with an AEFI were responsible for fear amongst caregivers and the reason provided for missing a scheduled vaccination on purpose. Two mothers explained their fear and anxiety as follows:

*“… Whenever you remember the pain your child went through in the last vaccination and the rise in temperature you get discouraged … so you then think the day you take your child for vaccination, you won´t sleep the whole night … so you decide to just avoid going again and sit around.”* (FGD1-P9; Single mother with primary education).

*“Once a child is vaccinated, that very night of vaccination, you cannot sleep … (since the child cries the whole night also) due to high fever after the administration of the first two doses of 6 weeks and 10 weeks DPT or having administered too many vaccines together”. The injections affect the child who then does not to sleep because of pain. It causes a lot of body heat and the injected leg cannot move.”* (FDG2-P4; married mother with secondary education).

***Communication challenges:*** communication challenges, especially around language barriers, made it difficult for caregivers to obtain the necessary information on AEFI or to verbalize their questions or concerns about possible adverse effects after vaccination. A single mother with a disability, who had not completed her school education, described her challenge in understanding English.

*“… For example, somebody like me who is a class two drop-out … and the doctors just talk a lot of English, English, English which I don´t understand so I decide to leave … so, the doctor sometimes too makes us to leave.”* (FGD4-2; single disabled mother school drop-out).

***Negative attitudes of HCWs:*** caregivers were cognizant that negative attitudes by HCWs towards them limit the reporting of AEFI. They reported that some HCWs were abusive, disrespectful, uncooperative and even careless with their response to questions or reports from parents concerning child immunization.

*“Some of the HCWs do not have respect … if you ask any questions, you will receive very harsh answers which you did not expect you to ask any question.”* (FGD7-P9; married mother with primary education working a saloonist).

***Inadequate information provided/concerns about AEFIs not addressed:*** participants further claimed that even if the HCWs responded to their concerns following immunization, in most cases the responses given were either unsatisfactory or fail to address the caregiver´s concerns.

*“… There are occasions when you take your child for the vaccine injection … but some doctors do not allow you to see the type of syringe they are using … and again sometimes you ask the doctor why the child has developed some marks and swelling and why he has to inject the same place, he/she tells you to leave the child alone …”* (FGD6-P11; married mother with secondary education and skilled worker)

**b) Reporting of AEFI by HCWs:** with regard to the reporting of AEFI by HCWs, awareness and expectancy of AEFI was evident during the KIIs, however a number of challenges with the reporting of AEFI by HCWs were verbalized.

***Uncertainty about vaccine contraindications and fear of blame for AEFI:*** most HCWs were dissatisfied with the narrowed list of conditions/diseases in the guidelines that were considered contraindications for vaccination. They admitted that it sometimes led them to misreport the reason for postponing a child´s vaccination e.g. exaggeration of a child´s condition in the child´s clinic card, to justify the reason for postponing vaccination. Furthermore, a number of HCWs elaborated on the fact that there was still concerns on how AEFI should be categorized, because HCWs were often blamed for AEFI.

*“Once a child is vaccinated, that very night of vaccination, you cannot sleep … since the child cries the whole night also due to high fever after the administration of the first two doses of 6 weeks and 10 weeks DPT or having administered too many vaccines together.” The injections affect the child who then does not to sleep because of pain. It causes a lot of body heat and the injected leg cannot move.”* (FDG2-P4; married mother with secondary education).

***Training needs of HCWs on AEFI:*** some prominently challenges regarding the identification and reporting of AEFI mentioned by HCWs were inadequate training, described as being few and not broad or detailed enough to enable them to correctly identify, manage and report AIFEs. One of the HCWs described the situation as follows, and also highlighted that the curriculum is outdated.

*“… the trainings majorly focus on the immunization the monitoring chart … to know the drop out and performance coverage … Almost the same with the first one although it gives percentage … again the curriculum was developed over 15 years ago … thus does not cover the current issues.”* (KI8).

The HCWs mentioned that these trainings are majorly delivered through seminars where a few selected representatives per facility attend and become trainers of trainees, or through continuing medical education at facility level. The evaluation of these trainings are through monthly reporting.

***Perceptions about the cause of AEFI:*** the majority of the key informants had experienced AEFIs, of which some of those mentioned included swelling and redness at the injection site, shock and fever. They identified the main causes of AEFIs mentioned as loss of potency of vaccine, the wrong vaccine given, expired vaccine, poor storage of vaccines (frozen or high temperatures) and the wrong injection site used.

*“I saw one case of a child with a big swelling that I thought was an abscess, after DPT”* (KI 4).

*“Yes, DPT is the main culprit, with high fever, redness, tenderness and swelling at the place of injection. When very severe, we advise the mothers to use paracetamol syrup and the child gets better* (KI 3).

***Caregiver education on AEFI:*** the need for caregiver education on AEFI was raised by HCWs in the KIIs. The only immunization education currently available for the public, is through morning health talks before immunization, which is mostly done by community health volunteers.

*“We need more education on the system for reporting these adverse reactions. The ministry has a system, though it seems it has not been standardized and not many nurses know it. Even how to record them and submit the reports is a problem. We need more education on it.”* (KI 5).

***Documentation for reporting AEFI:*** the majority of HCWs knew about AEFI reporting and the documentation to be completed. They were aware of the policy guideline requirements, but stated it does not exist in all facilities. They complained that reporting of AEFI requires excessive paperwork and effort, leading to inadequate reporting. This was further amplified by informal pressures not to report AEFI, as a result of the necessary documentation not being available, as illustrated by what one of the nurses said:

*“… I recently had a client with an AEFI … but I could not report because I did not have the reporting tools … but I gave medication for the same after consulting with the nurse in-charge … we followed up the child and it cleared.”* (KI4).

## Discussion

This study found a 12.3% rate of occurrence of AEFIs within the informal settlement in Nairobi (Figure 1). Three of these events were convulsions which warranted reporting as severe AEFIs. However, it is not known if these AEFIs were reported to the immunization program, as the study was not designed to verify transfer into practice. About one in every three children who experienced AEFIs was not brought back for their subsequent vaccinations. In our recent publication from a rural district in Uganda, the rate of AEFI occurrence was 41.2% [8]. This study, as in our earlier study, observed that incidents of less severe self-resolving cases of AEFIs may go unreported unless the HCW probes for them. Both studies, similar to what is reported across the continent, identified fever as the most common AEFI, with the rest being skin rash, convulsions, cough, swelling or redness at the injection site, irritability and refusal to breastfeed [7, 8, 10, 16, 17].

A review of the secondary data and records from the study facilities over the preceding 12 months revealed that no AEFI had been reported to any of the facilities. It is therefore not surprising that the study identified knowledge challenges amongst and the community in Mathare Valley on AEFIs, where to report them, to whom they should be reported and how to report them i.e. which tool and process to use. The Ministry of Health does have a system in place for the identification and the reporting of AEFIs, but this system has not been adequately implemented with the Nairobi county health care program. This is a big weakness in pharmacovigilance at the individual healthcare provider, health facility and sub-county levels. Healthcare workers are an important group of immunization stakeholders in the management of AEFIs [18], in the post-marketing surveillance of vaccines to assist in identifying any new adverse reactions that have not been described previously [19].

Most developing countries do not have robust and efficient pharmacovigilance systems in place, therefore, HCWs play an important role in this function, including safety and harmful aspects of medicines and vaccines [20]. A WHO vaccine safety database review in 2015 found that <1% of reports of AEFIs globally are from Africa, and even then, 97% of the African contribution emanated from only 10 countries, namely Zimbabwe, Egypt, South Africa, Ghana, Morocco, Democratic Republic of Congo, Tunisia, Sierra Leone, Nigeria, and Senegal [9]. The WHO Program for International Drug Monitoring (PIDM) started operating in 1968, but the first countries from Africa joined it in 1992, a number that had risen to 35 out of the 54 African countries by 30^th^ September 2015 [20]. Africa´s role in pharmacovigilance was delayed due to legislative delays, poor access to medicines, weak and poorly coordinated supply chains for medical supplies, inadequate knowledge on pharmacovigilance and inadequate human, financial and technical resources to support pharmacovigilance. Lessons from addressing these gaps need to be implemented to the area of adverse drug reaction reporting, and by extension the reporting and management of AEFIs [21]. A starting point is the implementation of the WHO´s Global Vaccine Safety Blueprint, which has 8 strategic objectives, namely detection of AEFI, adequate investigation of safety signals, use of appropriate tools and methods, adequate communication of vaccine safety issues, technical support and training, ensuring a regulatory framework is in place, global analysis and response and public-private information exchange [11].

This study found that most of the caregivers whose children experienced fever following immunization used paracetamol as an antipyretic and analgesic. Some previous reports documented health workers promoting the use of analgesics including ibuprofen and paracetamol just before the time of administration of some vaccines with the intention of controlling any pain, or fever, or inflammation that may follow the immunization [22]. Opinion remains divided about the possible negative implications of such interventions in terms of the effectiveness of the vaccines administered, with some studies suggesting that these analgesics may interfere with the antibody response of the child to the antigenic components in the vaccines [23]. However, there have been reports that given after the vaccination, these antipyretics may be beneficial, as seen from a systematic review, that found that the antipyretics reduce fever associated with vaccination and do not interfere with the antibody response of the body to the vaccine [24]. For some vaccines such as that to protect against meningitis B, usually given at 2 and 4 months, preemptive paracetamol administration soon after immunization is recommended, since this vaccine has been associated with very high fever that can cause febrile convulsions [25, 26].

Though the scope of this study was the 6-14 weeks scheduled vaccines, the caregivers in the FGDs who had older children reported cases of refusals for specific vaccines such as 18-month measles vaccine and the third dose of the pentavalent vaccine which they said were *“due to high fever after the administration of the first two doses or having administered too many vaccines together.”* These responses thus highlight the vital role of HCWs in promoting vaccination and providing correct accurate education about vaccines. HCWs also play an important role to improve the diagnosis and reporting of AEFIs and ensuring functional pharmacovigilance systems, it is important that caregivers, hence improve the management of AEFIs [19]. It is important that caregivers, local communities and HCWs are empowered and educated to effectively manage AEFIs, because this can in turn improve vaccination uptake and coverage [15, 27]. It is known that some immunization systems are not effective because of problems with inadequate knowledge, AEFIs and inappropriate vaccine handling (storage, cold chain related temperature fluctuations, transport and administration) [15, 16, 28-30]. There have been some reports that linked the death of 15 children in South Sudan to the use of vaccines damaged through insufficient and ineffective cold chain support [31]. Health workers need to be educated on the proper use of thermometers for temperature monitoring to ensure an efficient cold chain is maintained. Further, they should be trained on proper techniques and procedures for administration of vaccines, because when administered correctly, the risk of administration error-related adverse events is reduced [32].

The majority of the participants in this study considered immunization as an important strategy for child survival. However, there were still fear and concerns about side effects and AEFIs especially among the caregivers and some HCWs. It is worrying that a substantial proportion of those who experienced possible AEFIs did not attend the following scheduled immunization. Some health workers may fear that occurrence of AEFIs like abscess and swelling may mean they used the wrong administration technique and that they are to blame, making them feel guilty of causing harm [15]. HCWs were blamed by respondents for some AEFIs that occurred, and most caregivers were not sure about long-term adverse effects of vaccination. Most HCWs in Mathare Valley indicated that AEFI reporting required lots of paperwork from them, making them unwilling to report the adverse events accurately. As revealed in the focus groups, the main reported sources of negative information for vaccination were influence of misconceptions or myths, influence of friends or relatives and the fear of vaccine-related adverse effects. These were identified as major factors affecting acceptance of vaccines. It is indeed in agreement with increasing concerns that occurrence of AEFI could be contributing to rising vaccine hesitancy [8, 11, 28]. As deduced in the focus groups, some caregivers failed to take children for vaccination because of a previous experience of an AEFI in the child´s older siblings or among children of caregivers´ relatives/friends and in some cases the parents´ own experience with vaccination in their childhood, including fears of allergic reactions. These concerns need to be addressed, in order to build confidence among both caregivers and HCWs about vaccines and the possibility of occurrence of AEFIs.

This study had some important limitations and strengths. There was a likelihood of recall bias since caregivers were asked to recall whether their babies had experienced any untoward effects (AEFI) following the previous vaccine administered, which was 2-4 weeks earlier. Further, it was not possible to verify from the reports, which vaccine was responsible for many of the AEFIs since multiple vaccines were administered to the babies at each of the immunization visits that were covered by the study. We did not verify reporting rates of AEFIs beyond the study period, hence it is not possible to determine whether the project itself had an impact on subsequent AEFI reporting rates, and so it was not possible to determine the impact of the project on the AEFI reporting rates or whether the AEFIs that were identified in the study were reported appropriately to the authorities. A major strength of this study is that it highlights challenges around of vaccination in urban informal settlements, which is a unique population that can easily be forgotten. It also highlights an important topic in AEFIs that if addressed can help improve pharmacovigilance and reduce the overall impact of AEFIs on the rising cases of vaccine hesitancy. This study highlighted for the Nairobi county health department, to identify immunization areas that need priority improvement especially the need to design a simple and clear system for identifying, reporting and managing AEFIs, as well as disease surveillance and social mobilization for immunization. A positive outcome of this study is the training of 50 vaccinating HCWs and village health teams (VHTs) in the identification, reporting and management of AEFIs.

**Recommendations:** this study proposes the following interventions to address the gaps identified: training health workers with refresher/new courses on basics of immunization and identification, reporting and management of AEFIs, and on Communication and trust will be important to address e.g. using motivational interviewing; community sensitization about identification, reporting and management of AEFIs; implementing the Ministry of Health´s recommended system for identifying, reporting and management of AEFIs and designing and piloting a program that equips health workers and Village Health Teams (VHTs) to offer continuous education for care takers that runs along the static and outreach /mobile immunization sessions in the informal settlements. This can be done by providing enough information, education and communication (IEC) materials in the local languages.

## Conclusion

Reporting, documentation and management of AEFIs in Mathare Valley require improvement in the following areas: identification, reporting, treatment, building caregiver trust in HCWs and the immunization program, community education, social mobilization and involvement.

### What is known about this topic

- While all the currently recommended vaccines are considered safe and effective, these vaccines may occasionally lead to undesirable effects referred to as adverse events following immunization (AEFIs);
- AEFIs may reduce public confidence in immunization leading to reduced uptake of childhood immunizations and increased dropout rates, thus slowing down the gains made in ensuring universal access to all vaccines by all vulnerable populations;
- Rumors about, and fears of AEFIs, have been identified as some of the factors that contribute to failure to take children for immunization and for immunization dropout. It is not clear whether the problem is reporting or documentation of AEFIs by either the caregivers or healthcare workers, and there is limited information about the contribution of AEFIs on failure to complete the immunization schedule in Kenya.

### What this study adds

- The majority of the participants in this study considered immunization as an important strategy for child survival; however, there was still fear and concerns about side effects and AEFIs, especially among caregivers and some HCWs;
- It is worrying that a substantial portion of those who experienced possible AEFIs did not attend the subsequent scheduled immunization. HCWs were blamed by respondents for some AEFIs that occurred, and most caregivers were not sure about long-term adverse effects of vaccination;
- This study highlights that AEFIs occurrence could be contributing to rising vaccine hesitancy in urban informal settlements, the need to improve pharmacovigilance, the need to design a simple and clear system for identifying, reporting and management of AEFIs as well disease surveillance and social mobilization for immunization.
